# Femtosecond Pulse Ablation Assisted Mg-ZnO Nanoparticles for UV-Only Emission

**DOI:** 10.3390/nano10071326

**Published:** 2020-07-06

**Authors:** Anubhab Sahoo, Muralidhar Miryala, Tejendra Dixit, Alicja Klimkowicz, Bellarmine Francis, Masato Murakami, Mamidanna Sri Ramachandra Rao, Sivarama Krishnan

**Affiliations:** 1 Department of Physics, Indian Institute of Technology Madras, Chennai 600036, India; ph15d025@smail.iitm.ac.in (A.S.); tdixit@iiitdm.ac.in (T.D.); bellarmine@physics.iitm.ac.in (B.F.); 2 Superconducting Material Laboratory, Graduate School of Science and Engineering, Shibaura Institute of Technology, 3-7-5 Toyosu, Koto-Ku, Tokyo 135-8546, Japan; miryala1@shibaura-it.ac.jp (M.M.); masatomu@shibaura-it.ac.jp (M.M.); 3 Department of Electronics and Communication Engineering, Indian Institute of Information Technology Design and Manufacturing Kancheepuram, Chennai 600127, India; 4 Department of Engineering Science and Mechanics, Shibaura Institute of Techno.logy, 3-7-5 Toyosu, Koto-Ku, Tokyo 135-8546, Japan; alicja@shibaura-it.ac.jp

**Keywords:** femtosecond ablation, Mg-doped ZnO, nanoparticles synthesis, UV-emission, cathodoluminescence

## Abstract

The need for improved UV emitting luminescent materials underscored by applications in optical communications, sterilization and medical technologies is often addressed by wide bandgap semiconducting oxides. Among these, the Mg-doped ZnO system is of particular interest as it offers the opportunity to tune the UV emission by engineering its bandgap via doping control. However, both the doped system and its pristine congener, ZnO, suffer from being highly prone to parasitic defect level emissions, compromising their efficiency as light emitters in the ultraviolet region. Here, employing the process of femtosecond pulsed laser ablation in a liquid (fs-PLAL), we demonstrate the systematic control of enhanced UV-only emission in Mg-doped ZnO nanoparticles using both photoluminescence and cathodoluminescence spectroscopies. The ratio of luminescence intensities corresponding to near band edge emission to defect level emission was found to be six-times higher in Mg-doped ZnO nanoparticles as compared to pristine ZnO. Insights from UV-visible absorption and Raman analysis also reaffirm this defect suppression. This work provides a simple and effective single-step methodology to achieve UV-emission and mitigation of defect emissions in the Mg-doped ZnO system. This is a significant step forward in its deployment for UV emitting optoelectronic devices.

## 1. Introduction

Wide bandgap semiconductors have an immense potential for application in UV optoelectronics. Oxide semiconductors, such as ZnO, TiO_2_ and NiO are formidable contenders as active components in UV light sources [1]. In particular, ZnO is amongst the most versatile semiconductor platforms with potential applications in UV LEDs and even lasers. [2,3]. ZnO owes this popularity to its large bandgap, ease of doping [4] and its relatively large exciton binding energy, 60 meV, evident even at room temperature. While these features make the ZnO system an attractive choice for optoelectronics [5,6,7,8], they also render it an important candidate in photocatalytic, antibacterial and biomedical technologies [9,10,11]. The emission spectra of ZnO nanostructures are sensitive to their shape, size and defect content [8,12,13,14]. The ratio of near band edge emission (NBE) to deep level emission (DLE) reduces with a decrease in particle size due to surface defects [15,16], while morphology also influences the surface UV emission [12,13,14]. Doping is one of the most convenient techniques to systematically tune the emission properties of a semiconductor nanoparticle [17].

The singular advantage of this platform is the fact that the bandgap of ZnO can be easily engineered by introducing dopants into this host matrix, particularly in the form of metals with similar ionic radii such as Mg, Al, Cr and Ni to name a few [17,18,19,20,21,22]. Mg-doping in ZnO tunes the bandgap towards the UV region and induces higher exciton binding energies. This enhances its efficiency in light-emitting devices as well as sensors and increases the feasibility of utilizing this system for photo-catalysis and antibacterial activities [23]. As in the case of pristine ZnO, Mg-doped ZnO (Mg-ZnO) is also prone to defect emission. This pitfall requires appropriate attention to selectively enhance the UV-only emission in this doped oxide system. Particularly in the nanoparticle form, native surface defects introduce photoluminescence (PL) emission ranging from the UV to the visible light region. While these defects are undesirable for UV-only emission applications, they do find an application as fluorescent markers for bio-imaging [24]. Thus, a process which can control the occurrence and, therefore, the emission from these defects is pertinent. Our focus is on UV-only emission materials which are suitable for application in UV LEDs and lasers [2,3]. This goal is justified by the fact that such light sources capable of emitting in the entire UV range are important in communication, disinfection, and water purification [25,26,27].

Magnesium doping of ZnO can be achieved in several ways including sol-gel, solid-state doping, calcination at different temperatures, vapor phase transport and pulsed laser deposition (PLD) [24,28,29,30,31]. Nonetheless, pulsed laser ablation in liquid (PLAL) is ideally suited for the preparation of clean Mg-ZnO nanoparticles without unintended residue. Several pulsed laser based approaches have already been proposed and demonstrated. In reports employing nanosecond and picosecond laser pulses to prepare the ZnO nanoparticles [32,33] by ablation, the particle size could be manipulated using pulse energy. Such dependency was also reported in the case of femtosecond pulse ablation when Said et al. [34] demonstrated a particle size control while ablating a ZnO pellet. Using a similar technique, both the production of nanohybrids for biomedical applications [35] and the realization of Mg-doped ZnO with a shifted NBE peak [36] were realized. However, despite extensive exploration, the controlled tuning of NBE with respect to DLE, especially in a clean reagent-free one-step PLAL process, has neither been envisaged nor realized, to the best of our knowledge. In this article, we report, for the first time, facile control of this important optoelectronic characteristic in a single-step fs-PLAL process using the energy of ablating pulses as the knob to tune the ratio of NBE to DLE emission.

In this context, synthesis by pulsed laser ablation is remarkably efficient and convenient owing to the fact that it is an inherently top-down approach. Ablation using nanosecond laser pulses is frequently used, while much shorter femtosecond pulses are fast becoming candidates for the ablation of a target immersed in a liquid, fs-PLAL. This is, indeed, proving to be a facile route to produce Mg-doped ZnO. Chelnokov et al. [36] demonstrated the possibility of control over doping concentration, which led to a tuning of the NBE. In this work, the fs-PLAL method is shown to effectively produce ZnO and Mg-ZnO nanoparticles using the pure ZnO and 20% Mg-doped ZnO targets. The ablation was carried out using a beam of 500 fs laser pulses at different average powers in the range of 0.25–1.5 W, which is well above the ablation threshold of these targets [36]. The formation of doped nanoparticles was confirmed by X-ray diffraction (XRD), Raman spectroscopy and transmission electron microscopy (TEM). Using photoluminescence (PL) and cathodoluminescence (CL) spectroscopies, we demonstrate the tunability and control over the NBE emission of these nanoparticles. While, we observed a six-fold improvement in the NBE to DLE ratio of the PL spectra, remarkably, the CL emission from Mg-ZnO nanoparticles showed a stunning enhancement by a factor of 70. This PL and CL study brings new insights into the development of ZnO and doped ZnO systems for UV optoelectronics.

## 2. Results and Discussion

### 2.1. Structural Characterization

**Figure 1:**
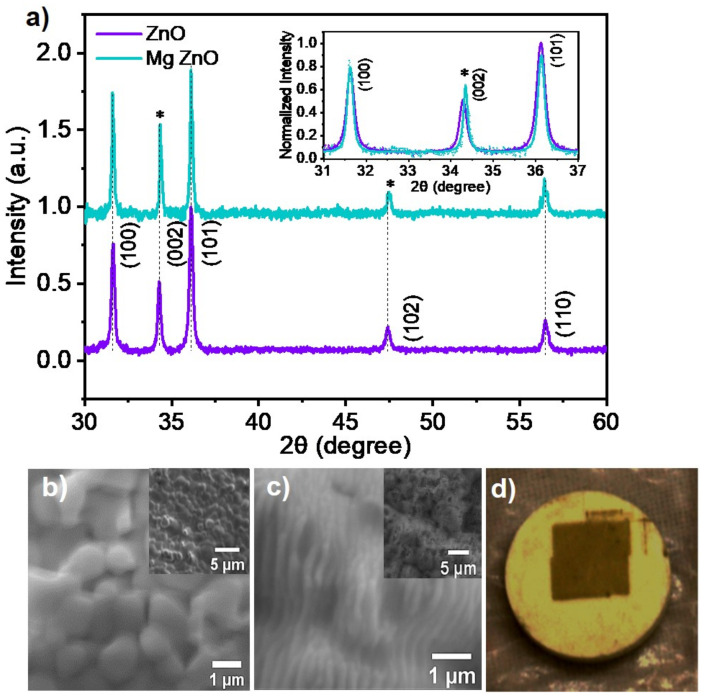
(**a**) The X-ray diffraction data of ZnO (violet) and Mg-doped ZnO (cyan) nanoparticles are compared here. The *inset* illustrates the peak shift between the two samples corresponding to the (002) plane. This evidences the presence of ${\mathrm{Mg}}^{2+}$ ions in the ZnO lattice replacing a fraction of the ${\mathrm{Zn}}^{2+}$ ions. Panels (**b**,**c**) are SEM images of regions of the pellet unexposed and exposed to the ablating fs pulses, respectively, shown in two different scales. This reveals quasi-periodic structures in the ablated region which are absent in the unexposed region (**b**), when both are imaged with the same magnification. (**d**) An image of the ZnO pellet used as the target in the fs-PLAL process. The light colored disk is the pellet prepared as described in the text. The dark patch inside this is the region where the target was ablated by fs laser pulses.

First, the doping of Mg in the ZnO matrix and the formation of Mg-ZnO nanoparticles has been confirmed by performing X-ray diffraction (XRD) measurements. A comparison of the XRD results for ZnO and Mg-doped ZnO (Mg-ZnO) nanoparticles are shown in Figure 1. The characteristic peaks of the hexagonal wurtzite structure of pristine ZnO are observed at 31.63°, 34.28°, 36.12°, 47.48° and 56.48° which have been indexed as (100), (002)^*^, (101), (102)^*^ and (110) planes referring to the JCPDS card no-036-1451 [37]. We observed shifts in the peak positions of the (002) and (102) planes for Mg-ZnO nanoparticles when compared to the pristine ZnO sample. These shifts were 0.06° and 0.05° for the (002)* and (102)* planes, respectively. These XRD results show no foreign peaks detected for pristine and Mg-doped ZnO specimens. This evidences the fact that high-quality doped nanomaterials were successfully produced in our single-step fs-PLAL implementation. Further, the lattice parameters *a* and *c* were calculated using the relation $a=\lambda /\sqrt{(}3)\sin\left(\theta_{100}\right)$ and $c=\lambda /\sin(\theta_{002})$ for angles corresponding to the (100) and (002) planes, respectively [29]. The calculated *a* value does not change after doping and is equal to about 3.26 Å, whereas a shift from 5.22 Å for ZnO to 5.21 Å for Mg-ZnO has been observed in the case of *c* parameter. Based on the work of Ohtomo et al. [38], this shift can be interpreted as arising from the ${\mathrm{Mg}}^{2+}$ ion substituting the ${\mathrm{Zn}}^{2+}$ ion in the ZnO lattice. Since the ionic radius of ${\mathrm{Mg}}^{2+}$ ion in IV coordination, 0.57 Å, is slightly smaller than that of the ${\mathrm{Zn}}^{2+}$ ion, 0.6 Å, there is a compression of the bond length in the $\left(\mathrm{Zn},\mathrm{Mg}\right)O_{4}$ tetrahedra [39]. The SEM image of unablated and ablated region of the ZnO pellet is shown in Figure 1b,c, respectively. The grain boundaries are clearly seen for the unexposed area, whereas the laser exposed area shows the formation of quasi-periodic structures following the interaction with fs pulses [40]. An image of the pellet used for laser ablation is presented in Figure 1d. The light-colored area represents the unexposed pellet, whereas the dark-colored patch is formed due to fs-laser irradiation.

**Figure 2:**
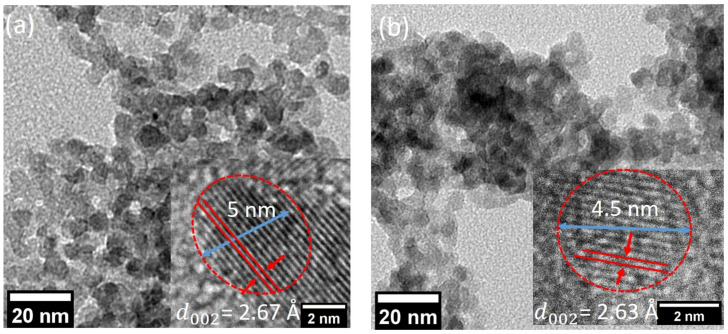
TEM images for (**a**) pristine ZnO and (**b**) Mg-doped ZnO. These nanoparticles were prepared using femtosecond laser pulses with an average power of 1.5 W. The *inset* in both panels shows the high-resolution TEM images from which the *d*-spacing was calculated to be 2.67 Å and 2.63 Å, respectively. These values are associated with (002) planes of the hexagonal wurtzite structure.

Transmission electron microscope (TEM) images of ZnO nanoparticles were recorded by drop-casting the suspension of nanoparticles in ethanol on a Cu mesh grid. These TEM micrographs are shown in Figure 2a for ZnO and Figure 2b for Mg-doped ZnO nanoparticles. In both cases, the particle size distribution was found to be within the range of 4–6 nm and the particles are spherical in shape. These high-resolution images allowed for the calculation of the *d*-spacing. The values were calculated by averaging over ten fringes to minimize errors and are equal to 2.67 Å and 2.63 Å for ZnO and Mg-ZnO, respectively. The obtained values correspond to the XRD data peak associated with the (002) plane (in the *d*-space scale). The *c* parameter can be calculated as twice the value of $d_{002}$ for a hexagonal structure. The calculated value of *c* is about 5.34 Å and 5.26 Å in the case of ZnO and Mg-doped ZnO, respectively. This indicates the contraction along the *c*-axis, which is in agreement with the XRD results. This reduction qualitatively confirms the substitution of Mg-atoms into the ZnO lattice [24].

### 2.2. UV Emission and Spectroscopy

**Figure 3:**
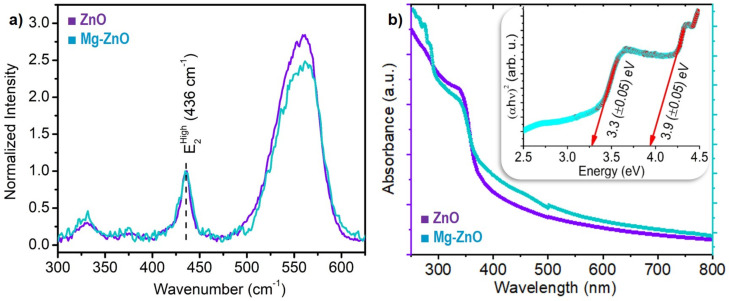
(**a**) Raman spectra of ZnO and Mg-doped ZnO nanoparticles synthesized by the fs-PLAL technique employing an average power of 1.5 W: These spectra were normalized at the $E_{2}^{\mathrm{High}}$ mode, 436 ${\mathrm{cm}}^{-1}$. (**b**) A comparison of the optical absorption spectra of undoped and doped ZnO nanoparticles. The *inset* is the Tauc plot for the Mg-ZnO nanoparticles evidencing the band-edge at 3.9(±0.05) eV in addition to the 3.3(±0.05) eV edge of pristine ZnO.

Raman spectra of ZnO and Mg-doped ZnO nanoparticles shown in Figure 3a were recorded using non-resonant 532 nm excitation. The results were gathered for ZnO (violet) and Mg-doped ZnO (cyan) nanoparticles prepared by fs-PLAL at an ablation power of 1.5 W. The Raman spectrum for the hexagonal wurtzite structured ZnO has two characteristic peaks at $99\;{\mathrm{cm}}^{-1}$ and $436\;{\mathrm{cm}}^{-1}$. These two peaks arise from the Zn and oxygen vibration modes, respectively, in the ZnO sub-lattice. The former is labelled as $E_{2}^{\mathrm{Low}}$ while the latter is $E_{2}^{\mathrm{High}}$ [41]. The $E_{2}^{\mathrm{High}}$ peak occurs at $436\;{\mathrm{cm}}^{-1}$ for both the ZnO and Mg-ZnO samples, indicating that the nanoparticles retained their wurtzite structure even after Mg-doping. These Raman spectra were normalized at the characteristic peak of 436 ${\mathrm{cm}}^{-1}$ and comprise of another broad peak in the 500–600 ${\mathrm{cm}}^{-1}$ region. The latter feature has contributions from two modes, namely, the surface optical phonon mode (SOP) and the $E_{1}\left(\mathrm{LO}\right)$ mode. These modes indicate the presence of both surface defects and oxygen vacancies in these nanoparticles [42] which are detrimental to UV and optical emission. Therefore, this part of the Raman spectrum gives a measure of surface defects in these nanoparticles prepared by the fs-PLAL process.

UV-Visible absorption spectra were measured for nanoparticles suspended in ethanol using a quartz cuvette with a path length of 10 mm. The absorption spectra of both ZnO and Mg-ZnO nanoparticle specimens prepared using fs-PLAL at 1.5 W are compared in Figure 3b. In both cases, absorbance drastically increases below 400 nm. However, in the case of Mg-doped ZnO a second band edge can be observed around 315 nm. The first band edge, in both spectra appears at ≈365 nm. Whereas for Mg-doped ZnO this is smooth, this edge is sharper in comparison for ZnO nanoparticles [43]. Using the Tauc plot [44], inset to Figure 3b, we decipher a clear edge at 3.9(±0.05) eV in the case of Mg-doped ZnO nanoparticles. This is in addition to the expected edge at 3.3(±0.05)eV corresponding to ZnO alone. The 3.9 eV edge in the case of Mg-doped ZnO follows closely the scaling law for ${\mathrm{Mg}}_{x}{\mathrm{Zn}}_{1-x}O$ systems compiled by Adachi et al. [45] based on works in thin films [46]. The additional shoulders seen in the spectrum may be attributed to defect states following the work of Tsay et al. [47].

**Figure 4:**
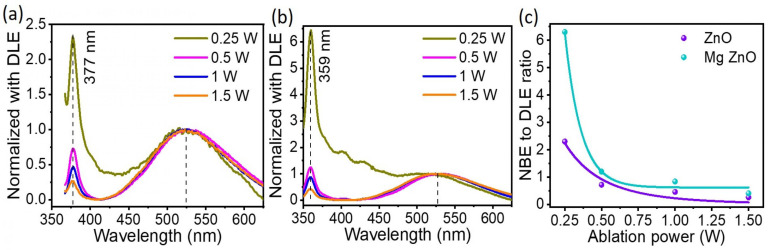
The photoluminescence (PL) for ZnO nanoparticles at room temperature for different ablation powers used in the fs-PLAL process, (**a**) for ZnO and (**b**) Mg-doped ZnO. PL emission from nanoparticles prepared with ablation powers of 0.25 W, 0.5 W, 1 W and 1.5 W are depicted in differently colored curves as indicated in the legend. (**c**) The ratio of the amplitudes of the NBE to DLE emission from fs-PLAL nanoparticles prepared and different average powers of the ablating beam.

The photoluminescence (PL) spectra of both nanoparticle systems undoped ZnO and Mg-ZnO prepared under otherwise identical laser pulses in the fs-PLAL process are compared in which a He-Cd laser at 325 nm was used to photoexcite them. Figure 4 shows the PL spectra in panel a) for ZnO and in panel b) for Mg-ZnO nanoparticles. These spectra are reported for a range of laser pulse parameters of the fs-PLAL process. Average fs-PLAL ablation powers of 0.25 W, 0.5 W, 1 W and 1.5 W were used identically in both cases to prepare four sets of pristine and doped nanoparticles for a systematic comparison. Their PL spectra were normalized at the DLE at 525 nm for a comparison of the near-band edge UV emission, 350–400 nm, which is of interest to us in this work. The NBE peak in the PL spectrum, which corresponds to the excitonic transition from the conduction band to the valence band, is blue-shifted by 18(±1.0) nm in Mg-doped ZnO with respect to ZnO nanoparticles, when both of them are produced in the fs-PLAL process with the same ablation power. The work of Chelnokov et al. [36] explored the systematic blue-shift of the NBE peak as a function of increasing Mg-doping. Based on this work, we estimate a lower bound of >10% for Mg-doping in ZnO in the fs-PLAL process. The NBE peak amplitude increases with respect to the DLE as the power of the ablating beam in the fs-PLAL process is lowered. For the lowest considered fs-PLAL ablation power (0.25 W), NBE enhancements of 2.5 fold and 6 fold for ZnO and Mg-ZnO, respectively, are evident when compared to nanoparticles prepared by a 1.5 W ablating beam. As the ablation power increases, the DLE emission amplitude increases due to increased defect formation. Thus, gentle fs-PLAL ablation at 0.25 W is ideal for realizing enhanced UV-only emission in Mg-doped ZnO nanoparticles. This is the hallmark of the present study.

We further analyzed the PL emission spectra of these nanoparticles by deconvoluting them to quantify the contribution of the participating energy levels. It is notable that the PL emission for both nanoparticles has similar features. The first of these is the characteristic sharp NBE peak in the 350–400 nm region, which is followed by the broad green DLE emission between 450–600 nm. The NBE can be attributed due to direct band-to-band transitions and energy levels close to the conduction band. However, an asymmetry at lower energies arises due to the contribution of zinc interstitials (${\mathrm{Zn}}_{i}$) [19,48]. We also note the identical blue-shift in the NBE for the case of Mg-doped ZnO nanoparticles irrespective of the ablation power used in fs-PLAL. This is clearly attributable to the content of Mg-doping, following earlier work [49]. The emission at 390–410 nm corresponds to transitions to the valence band from ${\mathrm{Zn}}_{i}^{+}$ and ${\mathrm{Zn}}_{i}^{2+}$ levels situated 0.22 eV and 0.5 eV, respectively, below the conduction band [37,48]. While the NBE of Mg-doped ZnO is blue-shifted with respect to pristine ZnO nanoparticles, the green emission in both cases is very similar, showing a broad symmetric feature center at ≈525 nm. This behaviour is due to the trap-assisted transition from donor level $V_{O}^{+}$ at 2.43 eV (510 nm) above the valence band [50], which is not influenced by doping [49]. The unchanged defect emission also indicates that there is no excess of Mg in the ZnO host, which may affect deep levels such as Mg-interstitials (${\mathrm{Mg}}_{i}$), as demonstrated by Trunk et al. [51]. The enhancement of UV emission with respect to the green emission in Mg-ZnO nanoparticles relative to ZnO is due to the strong bonding between Mg and O, which reduces the formation of oxygen vacancies [52]. Several reports evidence modifications to the PL emission spectra on account of their morphology and preparation mechanisms of ZnO nanomaterials: their size [15,16], their shape [12,16] and the process of synthesis [13,14,53] are known to affect the green DLE emission. The highlight of this work is that in a facile single-step synthesis process, fs-PLAL, we have demonstrated control over the NBE to DLE ratio of these Mg-ZnO nanoparticles using a single synthesis parameter, namely, the average power of the ablating beam. This adds immense value to the ZnO platform in realizing UV emission for practical applications.

**Figure 5:**
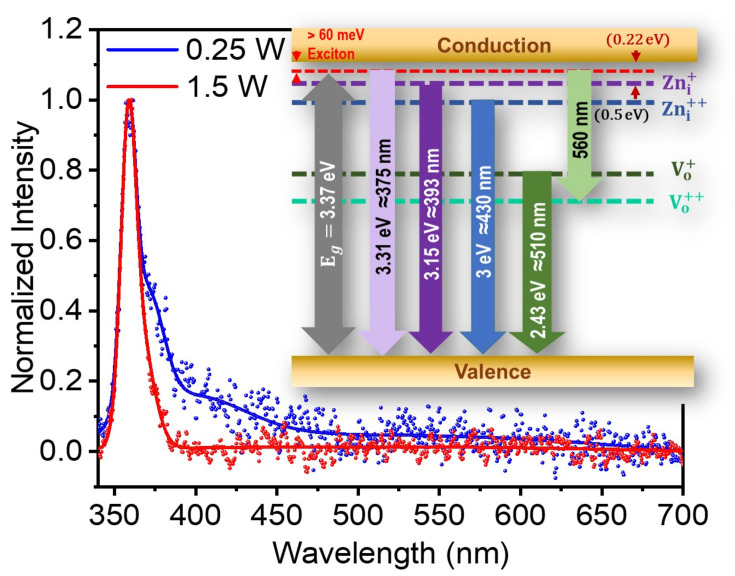
Cathodoluminescence spectra of Mg-doped ZnO nanoparticles. The specimens were prepared at two different ablation powers 0.25 W (blue) and 1.5 W (red) using a beam of 500 fs pulses in the fs-PLAL process. *Inset* The energy levels relevant for the discussion on the luminescent emission from ZnO systems are depicted including the valence and conduction bands, excitonic states, Zn-interstitials and oxygen vacancies.

Next, we recorded the cathodoluminescence (CL) spectra for Mg-ZnO nanoparticles after casting them on a Si substrate and by focusing a 15 keV electron beam on the specimen. The spectra registered for the doped nanoparticle sample prepared using two fs-PLAL conditions, ablation powers of 0.25 W, the minimum used, and 1.5 W, the maximum, are shown in Figure 5. Unlike photoluminescence, CL spectra show only UV emission with a sharp peak at 359 nm, otherwise identical in PL spectra.

The corresponding blue-shift in the case of the CL emission is 19(±1.0) nm, which is similar to the shift observed in PL spectra. The CL spectra were found to be highly influenced by the electron beam exposure time due to carbon adsorption on the surface layer, as reported by Jonas et al. [54]. The passivization of the surface layer leads to the quenching in the green emission, which originates from surface defects [55]. This also leads to an overall reduction in CL intensity. As expected, NBE dominates the luminescence in both the doped samples. However, when compared to the 1.5 W sample, the CL of Mg-ZnO nanoparticles prepared at 0.25 W of fs-PLAL ablation shows a marked emission towards longer wavelengths. This emission originates from the ${\mathrm{Zn}}_{i}$ levels below the band edge. It is also prominent in the case of the PL emission, as shown in the inset of Figure 5 [48]. When the ablation power is increased from 0.25 W to 1.5 W, the NBE to DLE ratio is enhanced from 25 to 70. This UV-only emission resulting from electron beam impact indeed is pertinent to applications optoelectronic devices which are electrically pumped.

## 3. Materials and Methods

Both pristine and Mg-doped nanoparticles were produced by femtosecond pulsed laser ablation in liquid, fs-PLAL. Ablation targets for pristine ZnO were prepared by finely grinding the ZnO powder and by pelletizing them with a hard-press at 5 MPa. These pellets were sintered at 800 °C for 24 h. To prepare targets for Mg-ZnO, a solid-state reaction method was employed to obtain precursors for pellets. Powders of ZnO and MgO were ground together, maintaining a molar weight ratio of 0.2:0.8 for 20% doping. Further, these admixtures were compressed and sintered at 1200 °C for 24 h. Thereafter, ablation was carried out using focused pulses Ti: Sapphire femtosecond laser (Astrella, Coherent Inc., Santa Clara, CA, USA) operating at a central wavelength of 800 nm with a repetition rate of 1 kHz. This laser beam focused on the target using a f= 200 mm converging lens. The target pellet kept at the bottom of a beaker containing 30 mL of ethanol with a liquid level of 30 mm maintained above the target surface to avoid contact with atmospheric air. This also ensured that the Rayleigh range of the ablating fs pulsed laser beam was entirely immersed in the solvent. This glass beaker containing the target was placed on a two-axis motion-controlled translation stage. This assembly was translated at a scanning speed of 300 μm/s, raster-scanning the target surface such that once ablated regions are moved away from subsequent laser pulses incident on the pellet. Thus, we ensured that a fresh region on the surface of the target was available for interaction with the ablating laser pulses. This is important to enable repeatability in the synthesis process as identical regions of the pelletized surfaces were available for each incident pulse in the laser-matter interaction taking place. The motion stages were programmed so as to have a length for each line of the raster-scan of 5 mm while moving in 100 μm steps. The entire ablation process lasted for 20 min for each iteration and was automated using a suitable program. In the ablation sequences, pulses with a full-width at half-maximum of 500 fs were used while the average power of the ablating beam was varied in the range of 0.25–1.5 W. The nanoparticles prepared in this fs-PLAL process were collected in the suspension contained in the glass beaker. Following the ablation process, these were transferred to sealed containers. An important post-ablation routine is the removal of inhomogeneous debris from the suspension containing the desired nanoparticles. In order to separate the debris, we centrifuged the as-obtained suspension at 2000 rotations per minute (RPM) for ≈15 min. The neat liquid containing only the suspended nanoparticles was extracted using a pipette leaving behind the unwanted debris at the bottom. This is a repeatable process for producing nanoparticle suspensions by fs-PLAL.

The crystal structure of the nanoparticles was measured in 2θ scans of X-ray diffraction (XRD) using a Rigaku Smartlab system. Further structural and microstructural characterization was carried out using a transmission electron microscope (TEM), JEM-2100F from JEOL, with a focused beam at 200 keV. For optical characterization, Raman spectroscopy, UV-visible absorption, photoluminescence (PL) and cathodoluminescence (CL) were performed. Raman spectra were acquired after drop-casting the nanoparticle suspension on a substrate in a WITec spectrometer under 532 nm excitation. The UV-Visible absorption and PL were measured while containing the suspension in a 10 mm quartz cuvette. A Horiba spectrofluorometer Flurolog with a double-stage grating pair monochromator was used in these PL measurements. CL measurements at room temperature were performed using a scanning electron microscope SSX-550 (Shimadzu) integrated with a cathodoluminescence attachment, from Gatan MonoCL3 with an HSPMT photomultiplier as the detector.

## 4. Conclusions

In this work, we demonstrate successful single-step facile production of Mg-doped ZnO nanoparticles by the fs-PLAL technique. These have excellent prospects for enhanced UV-only emission. As compared to pristine ZnO nanoparticles, the Mg-doped ZnO particles have a blue-shifted UV emission. While X-ray diffraction affirms the doping of Mg ions in the ZnO lattice, Raman spectroscopy evidences surface defects in these nanoparticles. By varying the average power of the beam of ablating pulses in the fs-PLAL process, control of these defects was achieved. At relatively low-ablation powers of 0.25W, the desired UV-only emission with very little accompanying green luminescence due to surface defects was successfully realized. This accomplishment is important from the viewpoint of applying nanoscale Mg-ZnO as the active material in realistic UV sources. Thus, our study is a forerunner to the further development of the scalable, easy and one-step synthesis of luminescent semiconducting oxide nanoparticles which can act as reliable, high-quality UV emitters ready for application.
